# Taxonomic updates in *Dolichandra* Cham. (Bignonieae, Bignoniaceae)

**DOI:** 10.3897/phytokeys.46.8421

**Published:** 2015-02-05

**Authors:** Luiz Henrique M. Fonseca, Simone Miranda Cabral, Maria de Fátima Agra, Lúcia G. Lohmann

**Affiliations:** 1 Departamento de Botânica, Instituto de Biociências, Universidade de São Paulo, Rua do Matão, 277, 05508-090, São Paulo, SP, Brazil; 2 Laboratório de Tecnologia Farmacêutica, Universidade Federal da Paraíba, 58051-059, João Pessoa, PB, Brazil

**Keywords:** Taxonomic key, Neotropical lianas, *Dolichandra hispida*

## Abstract

*Dolichandra* is a genus of lianas found in dry and wet Neotropical forests. The genus currently includes eight species and is well characterized by molecular and morphological synapomorphies. Here, *Macfadyena hispida* (DC.) Seemann is removed from synonomy with *Dolichandra uncata* (Andrews) L.G. Lohmann based on the presence of the hispid indument, vinaceus ovary, long fruits, and winged seeds. The combination *Dolichandra hispida* (DC.) L.H. Fonseca & L.G. Lohmann, **comb. nov.** is proposed, increasing the number of accepted species of *Dolichandra* to nine. A taxonomic key for all species of *Dolichandra* is presented.

## Introduction

*Dolichandra* Cham. is a genus of lianas that belongs to the tribe Bignonieae, in the plant family Bignoniaceae (Lohmann 2006; Lohmann and Taylor 2014). The family comprises ca. 80 genera and 840 species of trees, lianas and shrubs (Lohmann and Ulloa 2006, onwards), representing an important component of Neotropical forests and dry areas. The tribe Bignonieae includes 21 genera and 393 species of lianas and is centered in Brazil (Lohmann and Taylor 2014).

The current circumscription of *Dolichandra* is based on molecular phylogenetic data (Lohmann 2006) and morphological synapomorphies (Lohmann and Taylor 2014). In this circumscription, the genus is composed of eight species (Lohmann and Taylor 2014), one of which was previously included in *Dolichandra*, three in *Macfadyena* DC., one in *Melloa* Bureau, and three in *Parabignonia* Bureau ex K. Schum (Gentry 1973a, 1973b). Under the new circumscription, *Dolichandra* is characterized by unique multiple dissected phloem wedges, trifid and uncinate tendrils, fruits with four lines of dehiscence, a dimorphic growth form, a large and membranaceous calyx, and colpate pollen with a psilate exine (Gentry 1973a, 1973b; Gentry and Tomb 1979; Lohmann and Taylor 2014).

The genus is distributed in wet and dry Neotropical forests, from Mexico to northern Argentina (Gentry 1973a, 1973b; Lohmann and Taylor 2014), being a conspicuous component of seasonally dry forests. The geographic distribution of *Dolichandra* is centered in southern Brazil, northern Argentina and Paraguay, where up to seven species are found. The geographic distribution of members of *Dolichandra* is highly variable, with species found throughout the Neotropics, like the ubiquitous *Dolichandra unguis-cati* (L.) L.G.Lohmann, and species with restricted distributions such as *Dolichandra dentata* (K. Schum.) L.G.Lohmann, found in riverbanks of the Uruguay river basin (Lohmann and Taylor 2014).

*Dolichandra cynanchoides* (cham.) L.G.Lohmann is cultivated as ornamental in Argentina (García 1992) and *Dolichandra unguis-cati* in the USA (Gentry 1982). *Dolichandra unguis-cati* is also an invasive in some countries, like Australia and South Africa (Sparks 1999; Dhileepan et al. 2007). Attempts to reduce population size and control the invasiveness of *Dolichandra unguis-cati* are underway in both countries (Sparks 1999; Dhileepan et al. 2007).

During phylogenic and taxonomic studies of *Dolichandra*, it became clear that *Macfadyena hispida* (DC.) Seem. is morphologically distinct from *Dolichandra uncata* (Andrews) L.G.Lohmann and should be recognized as a separate taxon. We here present the necessary new combination. We also provide a taxonomic key for the genus, thus facilitating the identification of the species.

## Material and methods

This study was based on botanical collections from nine herbaria (ESA, FUEL, INPA, MBM, MO, NY, SP, SPF, and UPCB). Morphological studies were carried out under a stereomicroscope using dried and fresh specimens. Morphological terminology for leaves follows Hickey (1973) and flowers and inflorescences follows Weberling (1989). Other morphological structures follow Harris and Harris (2001).

## Taxonomic treatment

### Dolichandra hispida

Taxon classificationPlantaeLamialesBignoniaceae

(DC.) L.H.Fonseca & L.G.Lohmann comb. nov.

urn:lsid:ipni.org:names:77145082-1

Fig. 1

Spathodea hispida DC., Prodr. 9: 205. 1845.
Macfadyena hispida (DC.) Seemann, J. Bot. 1: 227. 1863. Type: Brazil. Mato Grosso: Cuiabá, 1832, A. Silva Manso 105A (holotype: G-DC [G00133604]!).
Spathodea mollis Sond., Linnaea 22: 561. 1849.
Macfadyena mollis (Sond.) Seemann, J. Bot. 1: 227. 1863. Type: Brazil. Minas Gerais: Caldas, 1855, A.F. Regnell I-292 (lectotype, designated here: MO [2229711]!).
Macfadyena pubescens Moore, Trans. Linn. Soc. London, Bot. ser. 2, 4: 418. 1895.

#### Type.

Paraguay. “inter Villa Maria et Corumbá”, Dec 1891–92, S. Moore 1021 (holotype: BM image [578432]!).

#### Description.

*Liana*. *Stems* terete, striate, interpetiolar region with ridges and glandular fields, eglandular and glandular trichomes covering the stem surface, eglandular trichomes simple, densely distributed in a hispid indument, glandular trichomes peltate and pateliform, flaky bark absent; prophylls 1.6–3 mm long, subulate, apiculate, smooth, hispid. *Leaves* bifoliolate with a terminal tendril; petioles semi-terete, hispid and with peltate trichomes, 0.95–4.49 cm long; petiolules terete, hispid with simple and peltate trichomes, 0.3–2.9 cm long, with equal length; tendrils trifid and uncinate; leaflets ovate, obovate or elliptic, apex acute to short acuminate with a drip tip, base rounded, symmetric or slight asymmetric, 3.2–8.6 × 1.2–7.14 cm, margin entire, membranaceous, the abaxial surface hispid with simple trichomes more concentrated on the veins, peltate trichomes throughout and pateliform glandular trichomes concentrated at the base, the adaxial surface hispid, primary venation straight, unbranched, secondary venation brochidodromous and tertiary venation percurrent. *Inflorescence* an axillary 3-flowered cyme, rarely reduced to one flower; pedicels 0.5–4.3 cm long, hispid and with peltate glandular trichomes; receptacle with pateliform trichomes; bracts deciduous, floral bracts filiform, deciduous, rarely present, elliptic to obovate, 0.7–5.5 mm long, membranaceous. *Calyx* green, bi-lobed, spathaceous with an incurved apicule, 1.4–3.2 × 0.7–1.6 cm, membranaceous, glabrate to hispidulous, with peltate trichomes. *Corolla* yellow, bilabiate with the upper 2 lobes reflexed and the lower 3 lobes forward, tubular-infundibuliform, glabrate, hispidulous 5.1–9.1 cm long, 1.3–2.2 cm, 4–5.7 cm wide; lobes obcordate, 1.2–2 cm long, 1.2–2.15 cm wide, margin entire. *Androecium* inserted at the tube, with simple trichomes at the insertion; short filaments 1.15–1.7 cm long, longer filaments 1.74–2.4 cm long, glabrous, attached at the same height from the base of the corolla, 4.5–9.4 mm from the base; staminode 8–9 mm long; anthers pale-yellow or white, 3–3.9 mm long. *Gynoecium* inserted at the tube, glabrous; pistil 3.3–3.8 cm long; ovary vinaceus, linear, 7–9 × 1.4 mm long; style 2.6–3 cm long; stigma rhombic. *Fruits* linear, attenuate toward base and apex, 77–125.8 × 1.17–2.2 cm, smooth, with lenticels, glabrous. *Seeds* with hyaline wings, thin, 2.2–3.8 × 1.4–1.8 cm wide, seed body oblong to ovoid, woody, 6–9 × 8–15 mm.

#### Nomenclatural notes.

A single specimen was cited by Sonder for *Spathodea mollis* labeled 292 in Regnell’s first series of collections from Brazil. Four specimens labeled as Regnell I-292 were located, one at K [000449792], two at BR [876279] [876378] and one at MO [2229711]. The best quality material is selected here as lectotype.

#### Taxonomic notes.

*Dolichandra hispida* is easily differentiated from all other species of *Dolichandra* by the unique hispid indument found on the vegetative and reproductive portions of this species, as well as the presence of a vinaceus ovary. *Dolichandra hispida* has been treated as a synonym of *Dolichandra uncata* since Gentry (1973a). However, the differences in indument (hispid vs. glabrous to puberulous), ovary color (vinaceus in *Dolichandra hispida* vs. green in *Dolichandra uncata*), and seed wing morphology (hyaline in *Dolichandra hispida* vs. woody in *Dolichandra uncata*) are clear, making the separation of these two species necessary. In addition, the difference in fruit length is also striking, with fruits being much longer in *Dolichandra hispida* (77–125.8 cm) than in *Dolichandra uncata* (9.2–38.5 cm). In fact, *Dolichandra hispida* presents one of the longest fruits of Bignoniaceae, and possibly one of longest capsules within the Angiosperms (Table 1).

**Table 1.** Comparison of *Dolichandra hispida* and *Dolichandra uncata*; non-overlapping characters are shown in bold.

Characters	*Dolichandra hispida*	*Dolichandra uncata*
Leaflet form	Ovate, obovate (rare elliptic)	Elliptic (rare Ovate, obovate)
Leaflet apex	Acute to short acuminate	Long acuminate (rare short acuminate)
**Indument**	**Hispid**	**Pubescent (rare glabrous)**
Calyx	Short apiculate (1.2–2.3 mm)	Long apiculate (1.8–3.4 mm)
**Ovary color**	**Vinaceus**	**Green**
**Fruit length**	**9.2–38.5 cm**	**77–125.8 cm**
**Seed texture**	**Hyaline**	**Corky/woody**

*Dolichandra uncata* occurs predominantly in riverbanks, swamps and mangroves, presenting seeds that are corky and supposedly adapted for water dispersal (Gentry 1973b). On the other hand, *Dolichandra hispida* is more common in non-flooded areas, presenting seeds adapted to wind dispersal.

#### Distribution.

*Dolichandra hispida* occurs in southern, southwestern and central Brazil, Paraguay and Bolivia, whereas *Dolichandra uncata* has its northern limit in Mexico and southern limit in Argentina and Uruguay (Fig. 2).

**Figure 1.**
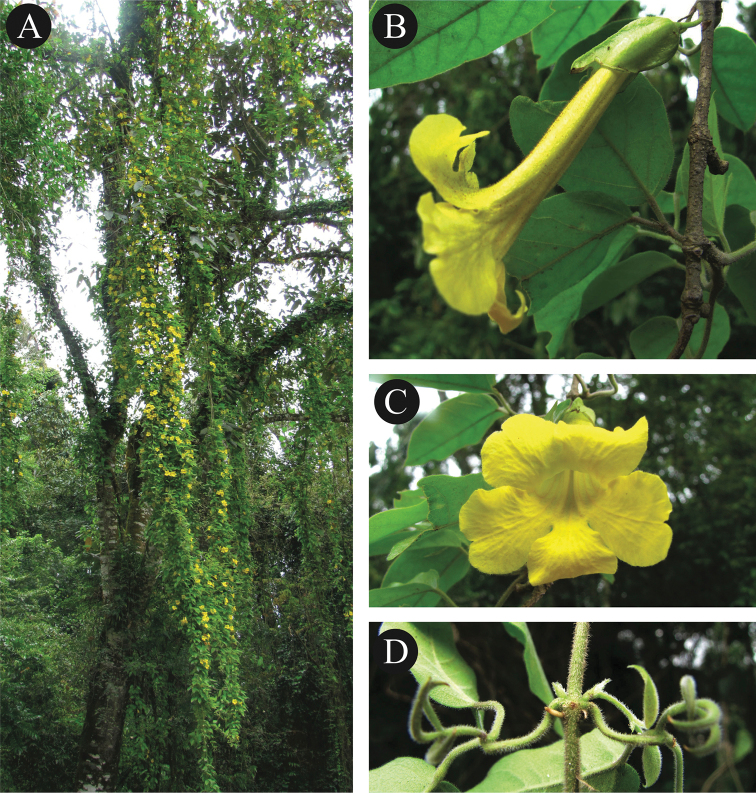
**A** Habit of *Dolichandra hispida* **B** Lateral view of flower **C** Flower frontal view showing the opening of the flower tube **D** Node branch showing the hispid indument (Photos L.H.M. Fonseca).

**Figure 2.**
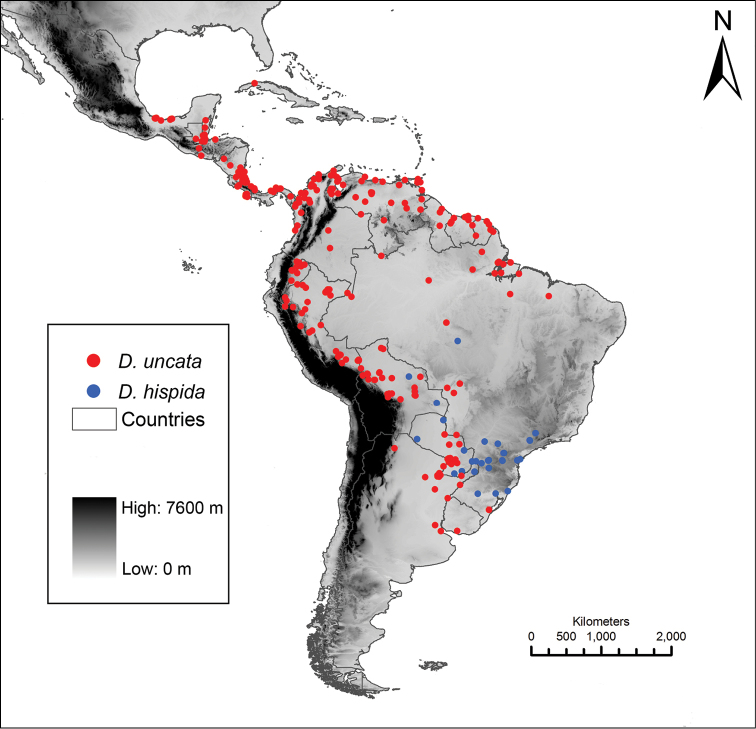
Distribution of *Dolichandra hispida* (red dots) and *Dolichandra uncata* (blue dots).

#### Phenology.

This species was collected in flower in September, October, November, December and January and in fruit in September, November, December, January and February.

#### Conservation status.

*Dolichandra hispida* is here considered as Least Concern [LC] according to IUCN criteria (IUCN 2012; IUCN Standards and Petitions Subcommittee 2014). The extent of occurrence estimated for the species is 2,209,625.833 km^2^ and the estimated area of occupancy is 875.000 km^2^ (cell width of 5 km). Therefore this classification was established based on the wide distribution of the taxon, since no population data is available.

#### Specimens examined.

**Argentina.** Misiones: Guarani, 03 Mar 2000, *N. Deginani 1630* (MO); **Bolivia.** Santa Cruz: Estancia San Miguelito, 200 km al E de la ciudad de Santa Cruz, 02 Dez 1996, *A. Fuentes 1342* (MO); Las Trancas, Lomerio, las parcelas de Bolfor, Las Trancas ‘95, 16 Nov 1994, *A. Jardim 1204* (MO); **Brazil.** Mato Grosso: Alta Floresta, Fazenda Mogno, Ponte do 27, margem direita, mata de capoeira, solo arenoso, 18 Sep 1991, *Macedo et al. 3009* (INPA); Paraná: Antonina, Rio Mergulhão, 31 Oct 1973, *G. Hatschbach 29172* (MBM); Foz do Iguaçu, Parque Nacional das Cataratas do Iguaçu, 14 Oct 1962, *G. Hatschbach 9378* (MBM); Guaraqueçaba, Tagaçaba de Cima, Rio Tagaçaba, orla da Floresta Atlântica, 20 Nov 2003, *G. Hatschbach et al. 76720* (MBM); Irati, Riozinho, 01 Oct 1982, *G. Hatschbach 45518* (MBM); Laranjeiras do Sul, Salto Santiago, 07 Mar 1991, *Silva et al. 955* (UPCB, SP); Fazenda Santa Ana, 31 Oct 1985, *Dias s. n.* (FUEL, MO); Morretes, início da Estrada do Itupava, beira do Rio Nhumdiaquara, próximo à ponte de Morretes, 29 m elev., 25°26'1.31"S, 48° 52'26.31"W, 12 Mar 2008, *L.H.M. Fonseca et al. 27* (SPF, MBM); Pinhão, Vale do Rio Iguaçu, Córrego Estreito, 22 Feb 1996, *G. Hatschbach et al. 64429* (MBM); Rio Bonito do Iguaçu, Fazenda Giacomet-Marodin, Pinhal Ralo, 23 Jun 1995, *Poliquesi & Cordeiro 328* (MBM, SPF); Tibagi, 696 m elev., 12 Oct 1959, *G. Hatschbach 6373* (MBM); Rio Grande do Sul: Morrinhos do Sul, Morro do Forno, trepadeira em borda de Mata Atlântica de encosta, 19 Oct 1997, *Jarenkow & Sobral 3204* (MBM); Santa Catarina: Apiúna, floresta ombrófila densa, 549 m elev., 27°10'27"S, 49°18'08"W, 11 Oct 2009, *K. Kniess 561* (SPF); São Paulo: Iporanga, estrada entre Apiaí e Iporanga, floresta ombrófila densa, próximo ao Rio Bethary, 240 m elev., 24°32'55"S, 48°41'09"W, 23 Oct 2010, *L.H.M. Fonseca & D. Tarabay 157* (SPF, SP, MBM, MO); Iporanga, estrada entre Apiaí e Iporanga, floresta ombrófila densa, próximo ao Rio Bethary, 23 Oct 2010, *L.H.M. Fonseca & D. Tarabay 162* (SPF, SP, MBM, MO). **Paraguay.** Chaco: Bahia Negra, 13 Nov 1946, *T. Rojas 13743* (MO); Itapua: Pirapo, Cerca Pirapo. Sitio de plantaciones experimentales de CEDEFO, 10 Oct 1984, *D.R. Brunner & W. Buck 853* (MO); Alto Parana: Puerto Stroessner, Itaipu, Forest Reserve Noe. Pto. Puente Stroessner, 27 May 1989, *A.H. Gentry 66144* (MO).

### Key to all species of *Dolichandra*

**Table:** 

1a	Calyx 5-lobed; branchlets with flaky bark; leaflets chartaceous	**2**
–	Calyx 2–3-lobed; branchlets without flaky bark; leaflets chartaceous or membranaceous	**3**
2a	Floral bracts linear–lanceolate to subulate, < 1 mm wide; calyx lobes rounded and shortly mucronate, magenta, puberulent; corolla puberulent outside with peltate trichomes at the lobes; Colombia, Costa Rica, and Ecuador	***Dolichandra steyermarkii***
–	Floral bracts elliptic or lanceolate, 2–3 mm wide; calyx lobes ovate–lanceolate, attenuate and mucronate, green, glabrous (except at margin); corolla glabrous outside (sometimes sparsely pubescent at apex); Brazilian Atlantic Forest	***Dolichandra unguiculata***
3a	Leaflet margins toothed; seed wings woody with a narrow hyaline margin; prophylls subulate, and smooth; riverbanks of Uruguay River basin	***Dolichandra dentata***
–	Leaflet margins generally entire (rarely toothed); seed wings hyaline, rarely woody but then, never with a hyaline margin; prophylls generally ovate and lanceolate, if ovate then striate, if smooth then lanceolate or subulate	**4**
4a	Anthers and stigma exserted; corolla bilabiate with the upper 2 lobes forward and the lower 3 lobes reflexed, red; fruit elliptic and coriaceous	***Dolichandra cynanchoides***
–	Anthers and stigma included; corolla bilabiate with the upper 2 lobes reflexed and the lower 3 lobes forward, yellow or purple; fruit linear, rarely elliptic, but then woody	**5**
5a	Leaflet chartaceous; calyx 3-lobed, covering approximately 1/3 of the corolla; corolla purple	***Dolichandra chodatii***
–	Leaflet membranaceous; calyx 2-lobed or truncated, covering approximately 1/4 or 1/5 of the corolla; corolla yellow	**6**
6a	Floral bracts foliaceous; calyx with a recurved apicule; fruit an oblong-elliptic capsule	***Dolichandra quadrivalvis***
–	Floral bracts filiform; calyx without an apicule, if apiculated then the apicule is incurved and never recurved; fruit a narrow, linear capsule	**7**
7a	Calyx cupular, truncate to sinuous, without an apicule; prophylls ovate and striate	***Dolichandra unguis-cati***
–	Calyx usually subspathaceously split, often with an incurved apicule; prophylls subulate-lanceolate or subulate and smooth	**8**
8a	Indument hispidous; ovary vinaceus; fruits 77–125.8 cm long; seeds with hyaline wings; deciduous forests of northern Argentina, southern, southwestern and central Brazil, Paraguay and Bolivia	***Dolichandra hispida***
–	Species glabrous to puberulous, never hispid; ovary green; fruits 9.2–38.5 cm long; seeds woody and opaque, hyaline wings absent; mangroves and swamps from Mexico to Argentina and Trinidad	***Dolichandra uncata***

## Supplementary Material

XML Treatment for Dolichandra hispida
